# Book Reviews

**Published:** 1897-03

**Authors:** 


### BOOK REVIEWS.

Illustrated Skin Diseases. An Atlas and Text-Book. With special reference to Modern Diagnosis and the most Approved Methods of Treatment. By William S. Gottheil, M.D., Professor of Venereal and Skin Diseases at the New York School of Clinical Medicine; Formerly Lecturer on Dermatology in the New York Polyclinic, etc., etc., etc. New York: E. B. Treat, 5 Cooper Union. Price per portfolio, $1.

We have received three portfolios of this work. The cover has the picture of a man with a camera. The sheets come to us simply folded and laid in the cover, perhaps with the intention that they be, ultimately, properly bound. The "Contents" are stated on the front of each portfolio, but each one runs without a definite stop into the next. About twenty-four pages compose a portfolio, above the four plates set in loosely.

The photogravures are excellent; the diagrammatic view of the skin is beautiful. The cuts from photographs are mostly poor. The text is written in plain sentences, but there is evidence of apparent haste, and the sacrifice of absolute accuracy and clearness to brevity—so often the fault in condensed works. For instance, he states that there are no nerves in the epidermis, while under "Nerves" he mentions the presence of nerve twigs, or fibrillæ in the rete. "Stratum spinosum," or "prickle cell layer" are not good synonyms, as given, for Rete Malpighii, or Mucosum. There are other kinds of cells, but other writers also misapply the term. The blood supply to the sebaceous glands is omitted. It is doubtful if one who examines a few mustached upper lips will agree that the hairs of the upper lip are implanted "straight." According to Duhring, Santi has shown that no cholesterin fats exist in the skin. All pustules do not have an areola, certain cachectic cases showing them seated on a pale skin. "Primary" and "Secondary" skin lesions are handled together. All syphilitic crusts are not rupia.

There are many interesting and practical points in the "General Etiology" and "General Therapeutics."

His insistence upon both constitutional and local treatment, the recognition of, and the ability to manage general conditions, is to be highly commended. However little we may find wrong with the general health, that "little wrong" must not be neglected for purely local treatment. Of course the doctor only favors internal treatment where there is something internal to treat.

He pictures and describes several dermal instruments and recommends a flat, side pressure instrument over the watch-key form of comedo extractor.

Without claiming perfection for it, he employs a modification of Jessner's classification.

Urticaria and Prurigo are put under the Oedemas—the sole representatives, while Quincke's disease is not mentioned. The class of Oedemas might, then, well be omitted—in the light of recent studies of urticaria and the presence of more fixed lesions in prurigo.

Fomulæ, in groups, are placed in the middle of many pages, generally as near as possible to the text referring to them. The formulæ are written with "parts" substituted for apothecary's or metric measures or weights, save No. 6; Asiatic pill. One formula is so given for internal use without directions as to the dose, number 30, p. 78, a printer's error, doubtless.

The author aligns himself with those who believe that the cholesterin fats penetrate the skin better than the glycerin fats.

His "General Methods" of treatment are very practical and interesting.

To return for a moment to the "Classification," epithelioma is not mentioned, but carcinoma appears, *only*, under new growths of the *glands*.

He adherres to the old Seborrhoeas in his description of diseases, so far ignoring either eczema or dermatitis, Seborrhoicum.

The description of the diseases, and the treatment is practical and easy of comprehension, the words being well chosen, and not used in redundance.

A picture of a giant comedo shows a large comedo projecting from a sebaceous cyst.

The criticisms which we have made are of the kindest. Many errors, or apparent errors, probably arose from haste, and having to make a positive statement, often with want of space for further elucidation. Again, what may appear erroneous to many dermatologists may yet be proven correct or undecided by many others.

The doctor has a clear style and states things with some confidence, rather than with the uncertainty characteristic of many who write books based upon the whole earth's literature upon the subject.

As a brief manual of instruction and a work of reference, Dr. Gottheil's book should be very popular. The work is doubtless not intended to occupy a field which the most ambitious authors have failed to cover. M. B. H.

---

A SYSTEM OF PRACTICAL MEDICINE. By American authors. Edited by Alfred Lee Loomis, M.D., late Professor of Pathology and Practical Medicine in the New York University, and William Gilman Thompson, M.D., Professor of Materia Medica, Therapeutics and Clinical Medicine in New York University. To be completed in four imperial octavo volumes, containing from 900 to 1,000 pages each, fully illustrated in colors and in black. Vol. I., Infectious Diseases. Per volume, $5.00. Lea Brothers & Co., Publishers, Philadelphia and New York.

The design of this System of Medicine presents a thoroughly practical work of ready reference for the practitioner of general medicine. Extended historical references and discussions of mooted theories have been omitted, but each author has sought to present the results of his personal experience and combine them with the views of other acknowledged authorities. In conformity with the practical character of this work, it will contain no general articles upon hygiene, bacteriology, pathology or symptomatology, but these subjects will be separately presented in connection with each disease, thus facilitating reference by making each article a complete practical treatise in itself. Much original research and investigation have been undertaken by the authors expressly for this work, the results of which the reader will find both in the text and in the illustrations. The latter have been made a special feature of those articles which admit of such elucidation. Minute details are given in each practical subject, as examination of the blood in malaria and in anemia, the examination of the sputa, physical diagnosis of the chest, the localization of disease of the brain, spinal cord, etc. Particular attention has been bestowed on full directions for treatment, while original prescriptions, formulæ, diagrams, charts and tables have been inserted wherever their admission seemed desirable.

This volume embraces the *Infectious Diseases:* Malaria, Dengue, Typhoid Fever, Typhus Fever, Yellow Fever, Relapsing Fever Cholera, Dysentery, The Plague, Influenza, Epidemic Cerebro-Spinal Meningitis, Erysipelas, Pyemia, Septicemia, Small-Pox, Varicella, Scarlet Fever, Measles, Diphtheria, Pertussis, Parotiditis, Tuberculosis, Syphilis, Leprosy, Tetanus, and Infectious Fevers of Obscure Nature. The articles are written by well known American physicians and teachers, and each monograph is complete and up to date. The article on Diphtheria (by Dr. W. H. Park, New York) highly commends the antitoxin, and more than twenty pages are devoted to a full discussion of this important therapeutic agent. The half-tone illustrations are excellent, particularly those on Malaria and Dysentery.

We predict for this System of Practice a marked and well deserved success.

---

THE DISEASES OF INFANCY AND CHILDHOOD. By L. Emmett Holt, A.M., M.D., Professor of Diseases of Children in the New York Polyclinic; Attending Physician to the Babies' Hospital, and to the Nursery and Child's Hospital, New York; Consulting Physician to the New York Infant Asylum. Illustrated with nineteen full-page colored photographic plates and one hundred and eighty-five cuts in the text. Sold only by subscription. Price, Cloth, $6.00. D. Appleton & Co., New York.

Unlike many of the elaborate systems which have flooded the medical market, the volume before us expresses the views of one man, and presents the results of his individual experience and research. The reader will appreciate the advantages of this. The book shows care in its preparation, an unusual simplicity of style, and a great amount of practical information which the practitioner needs in his daily care of infants and children.

The first two hundred and thirty-seven pages are devoted to the consideration of "The Care of the Newly Born Babe—Nutrition, Its Derangement and Diseases." From the standpoint of the practical physician this is the most valuable of the entire text. It is rich in useful information bearing upon these important subjects which play so important a part in the future welfare of the child. In the next great division of the text, "The Acute Diseases of the Lungs and Intestinal Tract," the material is not handled in quite as useful a manner as the preceding chapters; for it is here that we find cholera infantum placed as a synonym with acute gastro-intestinal infection and gastro-intestinal catarrh, and afterward handled as a separate and distinct disease. It is also in this that we find the dose of santonin for a child two years of age placed in excess of the amount which we would feel safe in administering. With these exceptions, this too is of great interest. The fourth division, "The Specific Infectious Disease," is treated in a thoroughly able manner, and is worthy of careful reading.

Taking the book in all its phases, it is one of the best which has been offered upon the subject of pediatrics, and should be read by all physicians interested in this important branch of medicine.

E. C. D.

---

ANOMALIES AND CURIOSITIES OF MEDICINE. By George M. Gould, M.D., and Walter L. Pyle, M.D. With 295 illustrations in the text, and 12 half-tone and colored plates. Price $6.00. W. B. Saunders, 925 Walnut street, Philadelphia.

This book will occupy an unique place, and it will supply a want which has doubtless been realized at times by all students of medicine. It is an "encyclopedic collection of rare extraordinary cases, and of the most striking instances of abnormality in all branches of medicine and surgery, derived from an exhaustive research of medical literature from its origin to the present day, abstracted, classified, annotated and indexed."

The work includes prenatal and genetic anomalies, obstetric anomalies, major and minor terata, anomalies of stature, size and development, physiologic and functional anomalies, surgical anomalies, anomalous diseases and instances of diseases, etc. The last chapter gives a short history of the principal historic epidemics. So far as we can discover, the book is remarkably free from typographical errors, as the works by this editor and this publisher generally are; on page 912, however, the date 1594 should be 1494.

The book is exceedingly interesting even for continuous reading, but it is as a work of reference that it will serve its most useful and important purpose. The profession should be grateful to the compilers of this book for the patience, energy and perseverance involved in bringing together in one volume this wonderful array of the anomalies and curiosities of medicine.

---

HIGH ALTITUDES FOR CONSUMPTIVES. By A. Edgar Tussey, M.D., Adjunct Professor of Diseases of the Chest in the Philadelphia Polyclinic; Consulting Physician to the Y. M. C. A. Gymnasium. Pp. 140. Price $1.50. Published by P. Blakiston, Son & Co., 1012 Walnut St., Philadelphia.

The full title of this book is the "Principles or Guides for a Better Selection or Classification of Consumptives Amenable to High Altitude Treatment and to the Selection of Patients who May be More Successfully Treated in the Environment to which They were Accustomed Previous to Their Illness," which expresses its scope and theory. We say *theory* because the author indulges more in general considerations and opinions than in the presentation of specific facts. He believes in the effects of high altitude on certain consumptive patients. "Any altitude more elevated than the one in which the patient has been a resident may, in a large proportion of cases be used as a therapeutic agent with the most pleasing results."

The author discusses some of the contra-indications to sending patients to elevated regions, such as serious constitutional disturbance, intense general bronchitis, small vital capacity, melancholy temperament, violent temper, sedentary habits, disparity between present condition and former standard of health, etc. The book contains some good ideas on muscle training, complications of consumption, alcoholism, etc. The author's style is somewhat light and ornate for a work of this character.

---

"DEUTSCH'S LETTERS." A Practical Method for Easy and Thorough Self Instruction in the German Language. Prepared with special regard to the close affinity existing between the English and German Languages. By Solomon Deutsch, A.M., Ph.D., author of Medical German; A Practical Hebrew Grammar, etc. Second and revised edition. Price $2. J. H. Vail & Co., New York.

This book has been written with a view to furnishing those desiring to learn the German language the means of doing so without the inconvenience and expense of a teacher. The instruction begins with a likening of the pure Anglo-Saxon words with the low German of 1300 years ago—yet spoken by the mass of country people in the lowlands on the Baltic. The letters and diphthongs and pronunciation are much more carefully taught than in other works of less scope.

Exercises in reading and pronunciation then follow, the German word, the corresponding English pronunciation, then the translation of the word. Instruction is then given in the writing of German in the German characters; the sentence, etc.

The grammar is intermingled with many definitions of words and multitudes of German-English exercises.

In short, the book appears to be most thorough, and, we think, one who masters it will have almost mastered the German language. M. B. H.

---

AMERICAN YEAR-BOOK OF MEDICINE AND SURGERY. By American Physicians, under the editorial charge of George M. Gould, M.D., Philadelphia. Profusely illustrated. 1897. Price $6.50. W. B. Saunders, 925 Walnut street, Philadelphia.

The cordial reception given the Year-Book for 1896 encouraged the editor and contributors to believe that the plan and execution of the work supplied a clear professional want.

The present volume is uniform in style and size with the Year-Book for last year, and shows upon examination the same judicious care in presenting the essential particulars of medical and surgical progress. One new and useful feature consists in short recapitulations of the trend and results of the year's work at the beginning of each department. Also, the plan of pronouncing editorial judgment upon new proposals and investigations has become a more distinctive characteristic in this volume. The index is unusually complete.

Physician's can find in these Year-Books all the important recent advances in the medical art.

---

THE MEDICAL ENVIRONMENT. By D. Campbell Black, M.D., Glasgow, Scotland. Published by Hugh Hopkins, Glasgow.

This little book (50 pages) contains two addresses lately delivered by the author, one "On the Hospital Question," the other "On Medical Ethics." The concluding paragraph of the last address will express his views: "I am satisfied, so far as concerns medical etiquette, with the Golden Rule. A gentleman, like a poet, is born, not made. The saints were the Lord's from the beginning. . . Emphatically, there should be no etiquette, and there can be no etiquette, in the medical or any other profession, other than the etiquette which regulates and controls the conduct of all men to who aspire to the moral dignity of gentlemen."

---

WE have received copy of the Transactions of the American Orthopedic Association for the tenth annual session, held in Buffalo, May 1896. The volume contains a large number of good papers relating to orthopedics, many of which are well illustrated. Among the illusrations are some finely executed skiagraphs of talipes, tubercular knees, rickety knock-knees, etc. The president of the Association is Dr. Samuel Ketch, of New York; the secretary Dr. John Ridlon, 103 State street, Chicago.

